# Selective Extraction of Sinapic Acid Derivatives from Mustard Seed Meal by Acting on pH: Toward a High Antioxidant Activity Rich Extract

**DOI:** 10.3390/molecules26010212

**Published:** 2021-01-03

**Authors:** Morad Chadni, Amandine L. Flourat, Valentin Reungoat, Louis M. M. Mouterde, Florent Allais, Irina Ioannou

**Affiliations:** 1 URD Agro-Biotechnologies Industrielles, CEBB, AgroParisTech, 51110 Pomacle, France; amandine.flourat@agroparistech.fr (A.L.F.); valentin.reungoat@agroparistech.fr (V.R.); louis.mouterde@agroparistech.fr (L.M.M.M.); florent.allais@agroparistech.fr (F.A.); 2 Extractis, 33 Avenue Paul Claudel, 80480 Dury, France

**Keywords:** sinapic acid, sinapine, ethyl sinapate, pH, mustard seed meal, extraction, polyphenols

## Abstract

The aim of this paper is to study the effect of the pH on the extraction of sinapic acid and its derivatives from mustard seed meal. Solutions of acidic pH (pH 2), basic pH (pH 12) and distilled water (uncontrolled pH ~ 4.5) were tested at different percentages of ethanol. The maximum extraction yield for sinapic acid (13.22 µmol/g of dry matter (DM)) was obtained with a buffered aqueous solution at pH 12. For ethyl sinapate, the maximum extraction yield reached 9.81 µmol/g _DM_ with 70% ethanol/buffered aqueous solution at pH 12. The maximum extraction yield of sinapine (15.73 µmol/g _DM_) was achieved with 70% ethanol/buffered aqueous solution at pH 2. The antioxidant activity of each extract was assessed by DPPH assay; the results indicated that the extracts obtained at pH 12 and at low ethanol percentages (<50%) exhibit a higher antioxidant activity than extracts obtained at acidic conditions. Maximum antioxidant activity was reached at pH 12 with buffer solution (11.37 mg of Trolox Equivalent/g _DM_), which confirms that sinapic acid-rich fractions exhibit a higher antioxidant activity. Thus, to obtain rich antioxidant extracts, it is suggested to promote the presence of sinapic acid in the extracts.

## 1. Introduction

Phenolic acids and their derivatives constitute one of the most common groups of phenolic compounds in plants. These molecules emerge as high value added products, as they play an essential role for human nutrition and health [1,2]. In recent years, sinapic acid and its derivatives such as sinapine and ethyl sinapate have attracted increasing attention thanks to their numerous biological activities. Indeed, sinapic acid has antimicrobial [3], antioxidant [4], antilipidemic [5], anti-inflammatory [6] and neuroprotective activities [7]. Moreover, sinapine has recently shown, thanks to its antioxidant activity, a protective effect against severe ischemic stress [8], and an inhibitory effect of acetylcholinesterase, beneficial for treating Alzheimer’s and Parkinson’s diseases [9,10].

Sinapic acid and its derivatives are present in various plants, especially in Brassicaceae seeds including rapeseed (*Brassica napus*) and mustard (*Brassica juncea and Sinapis alba*). Most of these phenolic compounds remain in the meal after pressing [11]. The amount of these compounds depends on the variety of the plant, the harvest year, the cultivation region and the processing method. Khattab et al. reported contents between 6.16–22.54, 17.73–19.71 and 14.56–16.66 mg/g of total phenolics in the defatted canola seeds (18 samples), press cakes (3 samples) and meals (7 samples), respectively [12].

Recently, the safety, side effects and the manufacturing process of chemically synthesized antioxidants have been discussed [13]. Thus, a need has arisen to replace them with bio-based antioxidant molecules, in particular in the pharmaceutical, cosmetic and food fields [14]. Industrial and agricultural by-products could be a source of these bio-based molecules. However, their production, which often includes an extraction and purification steps, must be relatively cheap. 

This paper deals with the valorization of mustard seeds meal, one of the main by-products generated by local mustard producers in the Grand Est region of France. These by-products represent an interesting and cheap renewable material for the obtention of high added value sinapic acid derivatives, thus providing a financial support to cultivate and promote these crops in the region.

The extraction process is considered the most critical step in sample preparation [15]. This applies to extracts rich in phenolic compounds which are isolated from biomass [16]. Organic solvents and/or physical pretreatments are generally used as they improve the extraction capacity of the process [14]. Concerning the extraction of sinapic acid derivatives, the most common process is the hydro-alcoholic liquid/solid extraction, either with ethanol [17,18,19] or methanol [20,21,22]. Other studies have investigated the combination of hydro-ethanolic extraction with physical pretreatments such as ultrasound [4,23,24], microwaves and high pressure accelerated solvent extraction [25].

An important aspect of the extraction of sinapic acid derivatives (SADs) is the selection of an appropriate pH. Indeed, several studies have shown that the pH affects the yield, stability and antioxidant activity of phenolic extracts [26,27,28]. The selection of the extraction pH depends on the chemical structure of the phenolic compounds and their pKa [29].

Mustard seeds meal being commonly acid (pH ~ 4.5), adjusting the pH of the extraction could influence the affinity of SAD compounds with the solvent. To the best of our knowledge, the influence of pH on the extraction of SADs has not yet been reported. Furthermore, in this work, the quantification of SADs is based on a precise measurement by UHPLC which is not the case in other studies where SADs were estimated through the measurement of total phenols by the Folin Ciocalteu assay [17].

Thus, the objective of this paper is to study and understand the effect of the pH of the extraction medium on the recovery of SADs from mustard seed meal. For this, the main SADs obtained from different extraction conditions will be identified by UHPLC. Then, the contents of the three main derivatives of sinapic acid (i.e., sinapine, ethyl sinapate and sinapic acid), and the antioxidant activity of the extracts will be measured for three pH (2, 4.5, 12) at different percentages of ethanol (0%, 30%, 50% and 70%). For basic pH solutions, the influence of three different alkaline agents used to reach pH 12 on the yield of extraction, the selectivity of extraction and the antioxidant activity was compared. 

## 2. Results and Discussion

Different operating conditions of the extraction process were tested for the extraction of SADs from mustard seed meal. The effects of the pH, the percentage of ethanol and the type of base used for the basification of the medium were determined. For this, the comparison of the UHPLC profiles, the determination of the SAD contents and the measurement of the antioxidant activity of the extracts were performed.

### 2.1. Comparison of the HPLC Profiles Under Different Operating Conditions

**Figure 1:**
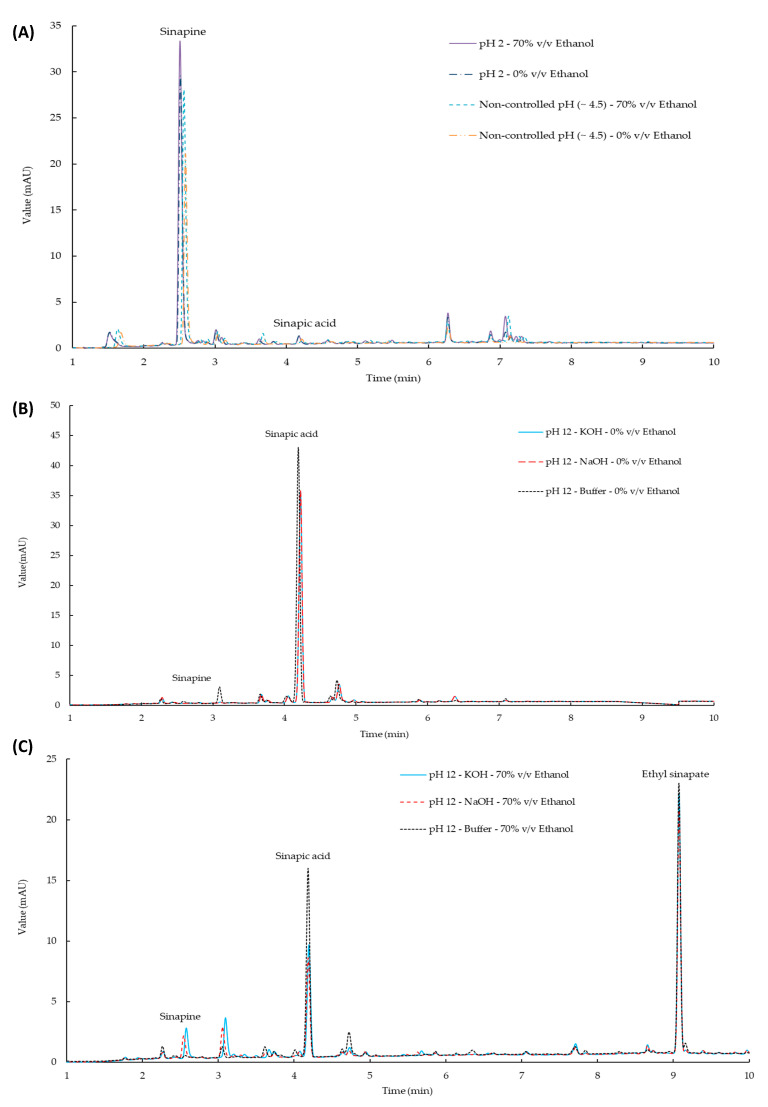
HPLC chromatograms of extracted sinapic acid derivatives (SAD) at acidic pH at 0% and 70% ethanol (**A**), basic pH at 0% (**B**) and basic pH at 70% ethanol (**C**).

The SADs extracted were identified by UHPLC analysis under different extraction conditions (pH, ethanol percentage). The chromatograms obtained are presented Figure 1.

Figure 1A shows the chromatograms of the extracts obtained under acidic conditions (uncontrolled (pH ~ 4.5) and pH 2) with 0% and 70% *v*/*v* ethanol. Sinapine is the main SAD that appears with a retention time of 2.5 min whereas sinapic acid is present in very low concentrations at 4.2 min. This result can be explained by the fact that, under these extraction conditions, sinapine is not hydrolyzed [30]. Thus, the amount of free sinapic acid that can be extracted from mustard seed meals is negligible as shown Figure 1A. Similar results were obtained by Dubie [4] during an aqueous extraction at a temperature of 80 °C from mustard seeds meal. The presence of ethanol in the extraction solvent increases the intensity of the sinapine peak, at acidic pH, without changing the selectivity of the extraction process.

The chromatograms of the extracts carried out at basic pH are shown in Figure 1B for an aqueous extraction (0% *v*/*v* ethanol) and in Figure 1C for a 70% *v*/*v* ethanol solvent. At basic pH, sinapine is present at very low concentrations, while the main SAD is sinapic acid for aqueous extraction (Figure 1B). This observation is consistent with previous results. Indeed, the formation of sinapic acid through the hydrolysis of sinapine is a very well-known reaction which occurs at basic pH or in presence of specific enzymes [31]. 

**Scheme 1:**
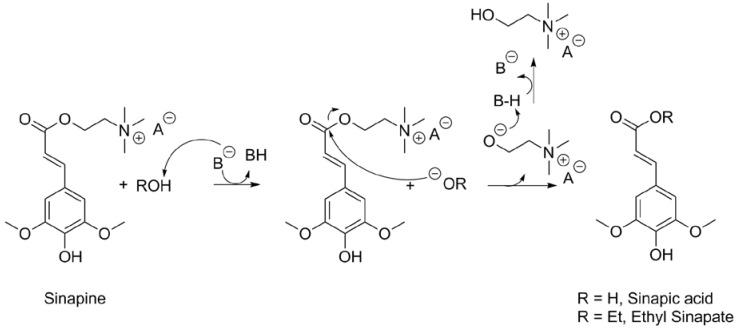
Reaction formation of sinapic acid and ethyl sinapate.

In the case of the hydro-alcoholic extraction (70% *v*/*v* ethanol) at basic pH, the most intense peak corresponds to ethyl sinapate with a retention time of 9.1 min. While ethyl sinapate can be formed through the esterification of sinapic acid in the presence of ethanol under acidic conditions, its production during the extraction process from mustard seeds meal in basic medium proceeds directly through the transesterification of sinapine by ethanol as shown in Scheme 1.

Moreover, an effect of the nature of the base on the intensity of the peaks is observed (Figure 1C). Indeed, when a buffer is used, an increase in the intensity of the sinapic acid peak is observed, whereas that of ethyl sinapate remains unchanged. Thus, the selectivity of the extraction process is improved by using a pH 12 aqueous (bis)carbonate buffer over KOH and NaOH solutions. 

### 2.2. Comparison of the SAD Contents Under Different Operating Conditions 

The comparison of the UHPLC profiles made it possible to show the selectivity of the extraction process on the SADs as a function of the pH and the percentage of ethanol. In order to quantify the effect of these two parameters, the contents of sinapine, sinapic acid and ethyl sinapate, as well as the antioxidant activity of the extracts, were determined.

#### 2.2.1. Evolution of the Contents of SAD at Acidic pH

**Figure 2:**
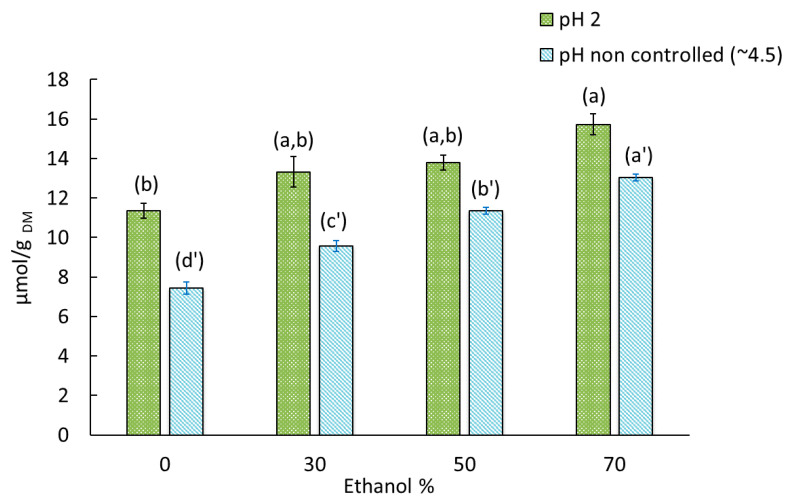
Evolution of the sinapine content from mustard seed meal at pH 2 and at non-controlled pH. Bars with the same letter are not significantly different (*p* < 0.05) according to Tukey’s test at the 95%.

As shown in Section 2.1, sinapine is the only SAD extracted at pH 2 and uncontrolled pH. The comparison of the sinapine contents obtained at pH 2 and at an uncontrolled pH for different percentages of ethanol is presented in Figure 2.

Extraction at pH 2 leads to higher yields of sinapine compared to the extraction at uncontrolled pH. The difference between these two conditions is particularly high for a low percentage of ethanol. Indeed, for an aqueous extraction (0% *v*/*v* ethanol), sinapine content increases by 52.5% between the two conditions, while the increase is only 20.7% for an extraction with 70% *v*/*v* ethanol. The highest yields of sinapine obtained are 15.73 and 13.03 µmol/g _DM_. They correspond to an extraction with 70% *v*/*v* ethanol at pH 2 and at uncontrolled pH, respectively. This is in agreement with the results of Reungoat [18] who found that the use of 70% *v*/*v* ethanol is the most efficient rate for sinapine extraction. Increasing the acidity of the extraction medium thus promotes the extraction of sinapine. This could be due to the denaturation of the cell membranes of the seeds, thus releasing the retained phenolic compounds. The same observation has been reported during the extraction of grape skin pomace under acidic conditions [32]. These results are in accordance with other researchers who reported 4.74 ± 0.28 mg/g _DM_, which is equivalent to 15.27 µmol/g _DM_ of sinapine [24], and 4–10 mg/g _DM_ of SAD with over 90% of sinapine in mustard seed meal [30].

#### 2.2.2. Evolution of the Contents of SAD at pH 12

**Figure 3:**
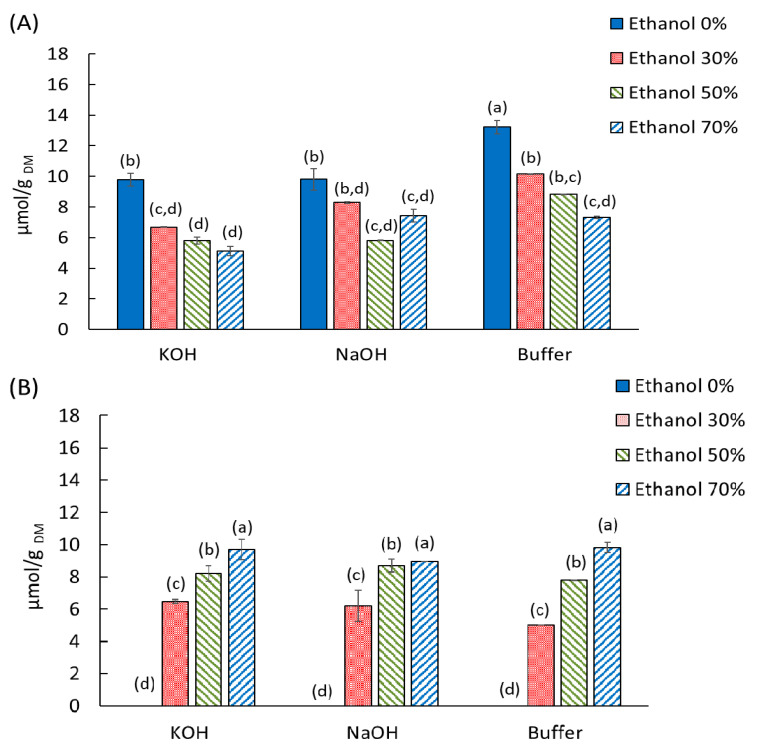
Sinapic acid (**A**) and ethyl sinapate (**B**) contents of extracts obtained at pH 12 and for different percentage of ethanol. Bars with the same letter are not significantly different (*p* < 0.05) according to Tukey’s test at the 95%.

Figure 3 presents the evolution of the sinapic acid (Figure 3A) and the ethyl sinapate (Figure 3B) contents for extraction at pH 12 with different percentages of ethanol and 3 alkaline agents. 

As shown in Figure 3, and as expected, the percentage of ethanol significantly (*p* < 0.05) affects the formation of sinapic acid and ethyl sinapate. The content of sinapic acid extracted is inversely correlated with the percentage of ethanol in the solvent. The sinapic acid content goes from 9.79 ± 0.03, 9.81 ± 0.69 and 13.22 ± 0.44 for an aqueous extraction to 5.13 ± 0.31, 7.43 ± 0.42 and 7.31 ± 0.06 µmol/g _DM_ with 70% *v*/*v* ethanol for the bases KOH, NaOH and buffer, respectively (Figure 3A). 

Figure 3A shows that the difference in yield of sinapic acid between a basified extraction with KOH and NaOH is not significant (*p* > 0.05). Concerning the buffer, no significant difference in yield exists with the previous two except for 0% *v*/*v* ethanol where the maximum yield of sinapic acid was obtained (13.22 ± 0.44 μmol/g _DM_). 

According to Figure 3B, the nature of the base does not affect the formation of ethyl sinapate. At 0% *v*/*v* ethanol, ethyl sinapate cannot be formed and by consequence was not detected. The amount of ethyl sinapate formed is only influenced by the percentage of ethanol in the extraction solvent. At 30%, 50% and 70% *v*/*v* ethanol, the yield in ethyl sinapate was 5.89 ± 0.59, 8.23 ± 0.31 and 9.49 ± 0.35 µmol/g _DM_, respectively. 

According to our results, aqueous extraction at alkaline pH gives significantly higher yields of total SADs than at acidic pH. The following classification could be established for an aqueous extraction of SADs: buffer pH 12 > pH 12 NaOH ≈ pH 12 KOH ≈ pH 2 > uncontrolled pH. The same classification can be made for an extraction at 70% *v*/*v* ethanol with an increase in SADs yield of 30%, 68%, 52%, 33% and 73%, respectively. Thus, one can conclude that increasing the percentage of ethanol had a significant effect on the total recovery of SADs. These results are in agreement with those reported by [4] who found that an extraction carried out with 70% *v*/*v* ethanol gives better yield of sinapic acid. This increase resulting from the addition of ethanol can be attributed to the change in the solubility, density or dielectric constant of the extraction solvent [33]. Moreover, the addition of ethanol could also accelerate the mass transfer between the liquid and the plant matrix by increasing the permeability of plant cell walls and help to break the bonds between solutes and the plant matrix [34].

### 2.3. Antioxidant Activity

**Figure 4:**
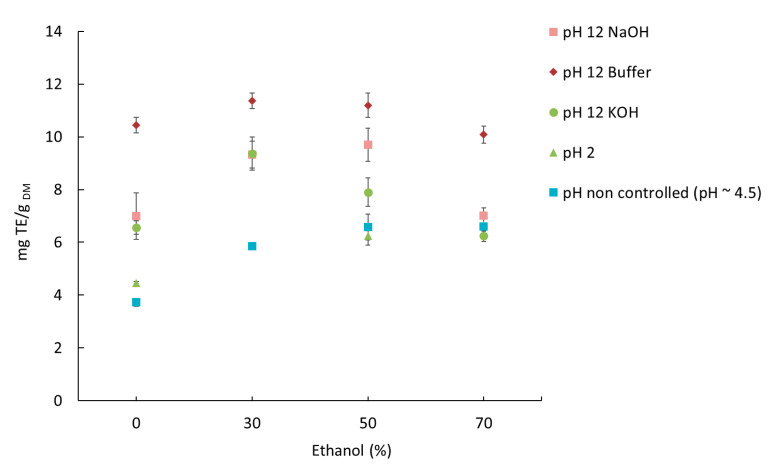
Antioxidant activity of extracts obtained at different operating conditions.

Several works have shown that SADs can be used as antioxidant molecules for different applications [3,35,36]. Figure 4 shows the antioxidant activity of the various extracts, measured by the DPPH radical scavenging method, expressed in mg of Trolox equivalent per 100 g _DM_. The SADs composition of the studied extracts is presented in the supplementary data section (Table S1).

According to Figure 4, the most antioxidant extracts are obtained at pH 12 with a buffer solution whatever the percentage of ethanol (*p* > 0.05). The antioxidant activity is 10.77 ± 0.5 g TE/g _DM_. The least antioxidant extracts are obtained for an acidic pH. This is explained by the presence of different SADs in the extracts. Indeed, at acidic pH, only sinapine is present in the medium, while at basic pH, the formation of sinapic acid occurs. This can be explained by a weaker antioxidant activity of sinapine (IC_50_ = 165.7 ± 0.9 μM) than that of sinapic acid (IC_50_ = 32.4 ± 2.2 μM) [37]. However, when ethyl sinapate is formed, a decrease in antioxidant activity is observed. This is the case for an extraction at 70% *v*/*v* ethanol, where all the antioxidant activities are equivalent except at pH 12 with a buffer solution. This can also be explained by the values of antioxidant activity. Indeed, ethyl sinapate has an IC_50_ of 51.9 ± 6.3 μM, whereas sinapic acid has an IC_50_ of 32.2 ± 6.2 μM [38]. Thus, to obtain the most antioxidant extracts from mustard seeds meal, it is necessary to promote the presence of sinapic acid in the extracts.

## 3. Material and Methods

### 3.1. Chemicals and Materials

Sodium hydroxide (97%), potassium hydroxide (85.3%), sodium bicarbonate (>99%), sodium carbonate (>99%), glacial acetic acid (99%), DPPH (2,2-Diphenyl-picrylhydrazyl) (>97.0%) and Trolox^®^ (6-hydroxy-2,5,7,8-tetramethylchromane-2-carboxylic acid)) (>98.0%) were obtained from VWR, Fontenay-sous-Bois, France. Acetonitrile (99.9%), formic acid (98–100%) and methanol (99.9%) were purchased from Thermo Fisher, Illkirch France.

Mustard seed meal (*Brassica juncea*) consisting of seed hulls, residual cotyledons and other minor seed fractions was used as the raw material. The mustard seed meal was obtained as by-product of the mechanical cold pressing of cured mustard seeds and was provided by Charbonneaux-Brabant (Reims, France) and stored in the dark at 4 °C until extraction.

### 3.2. Extraction Process

Extraction of SADs from mustard seed meal was performed in a 250 mL tricol flask with a condensing column. The solvent used is water at different pH and absolute ethanol at different percentages (*V*_Ethanol_/*V*_Water_), the liquid-to-solid ratio was fixed at 10 mL/g of seed meal. The temperature of extraction was fixed during the extraction at 70 °C in order to avoid the evaporation of ethanol and to provide better extraction yield as shown in our previous papers where temperatures between 70 and 75 °C were found to be better for the extraction of sinapic acid derivatives [17,18]. Time of extraction was optimized at 0% ethanol in non-controlled pH solution, it was found that the extraction kinetic was stable after 2 h, then the time of extraction was fixed at this duration.

The different experiments performed to study the effect of the pH on the extraction process are gathered in Table 1.

Carbonate–bicarbonate buffer (0.1 M) was prepared at pH 10.6 from sodium bicarbonate and sodium carbonate and adjusted to pH 12 by a solution of 1 M NaOH. Basic solutions of NaOH and KOH were prepared by adjusting the pH of distilled water with 1 M solutions of sodium hydroxide and potassium hydroxide, respectively. Acidic solution was prepared by adjusting the pH to 2 by adding glacial acetic acid (99%) to distilled water. For uncontrolled pH, the mustard seed meal was used as received from the company without any modification, the value of pH at 10 g/mL in distilled water was 4.5.

The liquid extract was separated from the solid residue by centrifugation (Allegra X15-R, Beckman Coulter, Villepinte, France) at 4713 g for 20 min at 4 °C.

### 3.3. Ultra High-Performance Liquid Chromatography (UHPLC) Analyses

The extract obtained from mustard seeds meal was filtered through a 0.20 μm, Chromatofil filter, Xtra RC-20/25, with a 1-mL syringe. Sinapine, sinapic acid and ethyl sinapate were quantified by reversed-phase UHPLC-DAD (Ultimate 3000; Dionex, ThermoFisher, Illkirch, France) equipped with a quaternary pump, auto sampler, column furnace and diode array detector. A gradient elution was performed using water (solvent A), acetonitrile (solvent B) and formic acid 0.1% (solvent C) on a C18 Thermo Scientific™ Accucore™ aQ; 100 × 3 mm with 2.6 μm particle size. Initial solvent was 45% A, 5% B and 50% C. Solvent B gradient followed: 5% (0 min), 10% (0.990 min), 15% (3.190 min), 30% (7.440 min), 5% (8.510 min), while C remained constant. The column was maintained at 48 °C and run at a constant flow rate of 0.8 mL/min. Total run time was 13 min with a 20 μL injection volume, and the detection wavelength was 320 nm. Sinapine chloride and ethyl sinapate standards were synthesized in-house and used to establish calibration curves [3]. Sinapic acid standard (≥98%) was purchased from Sigma-Aldrich, Saint-Quentin-Fallavier, France. NMR spectrum of sinapine and ethyl sinapate are presented in the supplementary material section.

### 3.4. Antioxidant Activity Assay

The radical scavenging activity was determined using a solution of 2,2-Diphenyl-picrylhydrazyl (DPPH•) at 0.06 mM. The antioxidant capacity of a given molecule was measured against a standard, Trolox (6-hydroxy-2,5,7,8-tetramethyl-chroman-2-carboxylic acid). The scavenging capacity was determined by monitoring the decrease in absorbance at 515 nm (maximum absorbance of DPPH•), which disappeared upon reduction by an antiradical compound. Spectrophotometric analyses were carried out as described by Brand-Williams [39]. For the determination of the radical scavenging activity, 1450 µL of DPPH• colored radical (0.06 mM in 96% methanol) was added to 50 μL of a suitable dilution of sample extract or Trolox (standard) in methanol solution. The mixture was stirred and then incubated in the absence of light at room temperature for 1 h. Absorbance was measured at the wavelength of 515 nm [39]. The percentage of DPPH• inhibition was calculated using Equation (1):

(1) $$Inhibition\;\left(\%\right)=\;\frac{A{\;}_{Control}-\;A_{\;Sample}}{A{\;}_{Control}}\;\times 100$$

where $A{\;}_{Control}$ is the absorbance of the control at 515 nm, and $A{\;}_{Sample}$ is the absorbance of the sample at 515 nm.

Methanolic solutions of Trolox at 0.05 (0.0125 mg/mL) to 1 mM (0.25 mg/mL) were used for calibration. The radical scavenging activity was expressed as mg of Trolox Equivalent (TE) per 100 g of dry matter (mg TE/100 g _DM_).

### 3.5. Statistical Analysis

All experiments were conducted at least in duplicates. The error bars indicated on the figures represent the standard deviations. Significant effects of pH and ethanol percentages were determined by an ANOVA (*p* < 0.05), and a Tukey test was carried out to find significative differences between groups. Statistical analysis was performed under R [40].

## 4. Conclusions

This study highlights the importance of controlling the operating conditions of the extraction process for the recovery of SADs from mustard seed meal. Indeed, it has been shown that at acidic pH, the main SAD is sinapine, while at basic pH, in the absence of ethanol, sinapic acid is mainly present. Moreover, in the presence of ethanol, at basic pH, a sinapic acid/ethyl sinapate mixture is obtained. The ethyl sinapate content is also maximum at pH 12 with a 70% *v*/*v* ethanol/buffer solution.

The use of SADs from mustard seed meal as a natural antioxidant will boost the substitution of synthetic antioxidants by natural antioxidants, since synthetic antioxidants have been shown to have negative health effects, such as lipid, enzyme and pathological alterations. In this work, it was found that extraction at basic pH with a buffer solution maximizes the antioxidant activity of the extract by increasing the selectivity of the extraction process for sinapic acid. Extracts with high ethyl sinapate content have shown slightly lower antioxidant activity than sinapic acid rich extract and higher than sinapine rich extracts. Ethyl sinapate rich extracts could be more advantageous for antioxidants intended to function in hydrophobic media such as membrane cells.

In a concept of valorization of mustard seed meal, techno-economic calculations will have to be made in order to determine the economic viability of such a process. Testing extraction at non-controlled pH was important in order to evaluate the effectiveness of extraction from crude seed meal without any previous modification of pH, which will reduce the cost and the environmental impact of the industrial process. Typically, small pilot-plant scale experiments need to be performed for validation and further optimization of the process conditions.

## Figures and Tables

**Table 1:** The different operating conditions tested for the extraction of SADs.

pH Medium		pH Value	Time (h)	Temperature (°C)	Ethanol (*v*/*v*%)
Acid		2	2	70	0
					30
					50
					70
Base	Sodium Carbonate-bicarbonate (NaHCO_3_/Na_2_CO_3_) buffer	12	2	70	0
					30
					50
					70
	NaOH	12	2	70	0
					30
					50
					70
	KOH	12	2	70	0
					30
					50
					70
Uncontrolled (pH of the mustard seed meal)		~4.5	2	70	0
					30
					50
					70

## Data Availability

Data is contained within the article or supplementary material. The data presented in this study are available in this manuscript.

## Supplementary Materials

The following are available online, Table S1: SADs composition of the studied extracts; Figure S1: ^1^H-NMR spectrum of sinapine; Figure S2: ^13^C-NMR spectrum of sinapine; Figure S3: ^1^H-NMR spectrum of ethyl sinapate; Figure S4: ^13^C-NMR spectrum of ethyl sinapate.
