# Supplementary material for: Selective Extraction of Sinapic Acid Derivatives from Mustard Seed Meal by Acting on pH: Toward a High Antioxidant Activity Rich Extract
Source: Molecules. 2021 Jan 3;26(1):212. doi: 10.3390/molecules26010212 (PMC7795640; doi:10.3390/molecules26010212)
Supplement: Supplementary file 1 [file molecules-26-00212-s001.pdf]

Supplementary Materials

Selective Extraction of Sinapic Acid Derivatives from Mustard Seed Meal by acting on pH: Toward a High Antioxidant Activity Rich Extract

Morad Chadni[*,1], Amandine L. Flourat[1], Valentin Reungoat[1,2,] Louis M. M. Mouterde[1], Florent Allais[1], Irina Ioannou[*1]

[1] URD Agro-Biotechnologies Industrielles, CEBB, AgroParisTech, 51110 Pomacle, France

[2] Extractis, 33 avenue Paul Claudel, 80480 Dury, France

* Correspondence: morad.chadni@agroparistech.fr (M.C.); irina.ioannou@agroparistech.fr (I.I.)

Supplementary Materials and Methods

NMR analyses were recorded on a Bruker Fourier 300. ^{1}H NMR spectra of samples were measured on a 300 MHz apparatus at 25 °C; chemicals shifts were reported in parts per million relative to solvent residual peak (CDC_{l3} δ = 7.26 ppm; CD3OD, 4.87 ppm). ^{13}C NMR spectra of samples were recorded at 75 MHz at 25 °C and calibrated on solvent peak (CDC_{l3} δ = 77.16 ppm; CD3OD, 49.2 ppm).

Supplementary Table and Figures

Table S1 : SADs composition of the studied extracts

Ethanol (%v/v)	pH medium	Sinapine (mg/g DM)	Sinapic Acid (mg/g DM)	Ethyl Sinapate (mg/g DM)
0%	pH 12 NaOH	N.D	9.81 ± 0.69	N.D
	pH 12 Buffer	N.D	13.22 ± 0.44	N.D
	pH 12 KOH	N.D	9.79 ± 0.03	N.D
	pH 2	11.35 ± 0.37	0.08 ± 0.04	N.D
	pH non-controlled	7.44 ± 0.3	0.1 ± 0.08	N.D
30%	pH 12 NaOH	N.D	8.29 ± 0.07	6.20 ± 0.96
	pH 12 Buffer	N.D	10.15 ± 0.02	5.00 ± 0.04
	pH 12 KOH	N.D	6.68 ± 0.02	6.46 ± 0.12
	pH 2	11.32 ± 0.77	0.05 ± 0.01	N.D
	pH non-controlled	9.57 ± 0.28	9.57 ± 0.29	N.D
50%	pH 12 NaOH	N.D	5.80 ± 0.02	8.70 ± 0.42
	pH 12 Buffer	N.D	8.28 ± 0.01	7.78 ± 0.03
	pH 12 KOH	N.D	5.81 ± 0.22	8.21 ± 0.49
	pH 2	11.78 ± 0.38	0.08 ± 0.01	N.D
	pH non-controlled	11.35 ± 0.17	0.07 ± 0.03	N.D
70%	pH 12 NaOH	N.D	7.43 ± 0.42	8.97 ± 0.05
	pH 12 Buffer	N.D	7.31 ± 0.06	9.81 ± 0.31
	pH 12 KOH	N.D	5.13 ± 0.31	9.69 ± 0.61
	pH 2	15.73 ± 0.54	0.08 ± 0.03	N.D
	pH non-controlled	13.03 ± 0.17	0.07 ± 0.04	N.D

N.D : non-determined

Figure S1 : ^{1}H-NMR spectrum of sinapine.

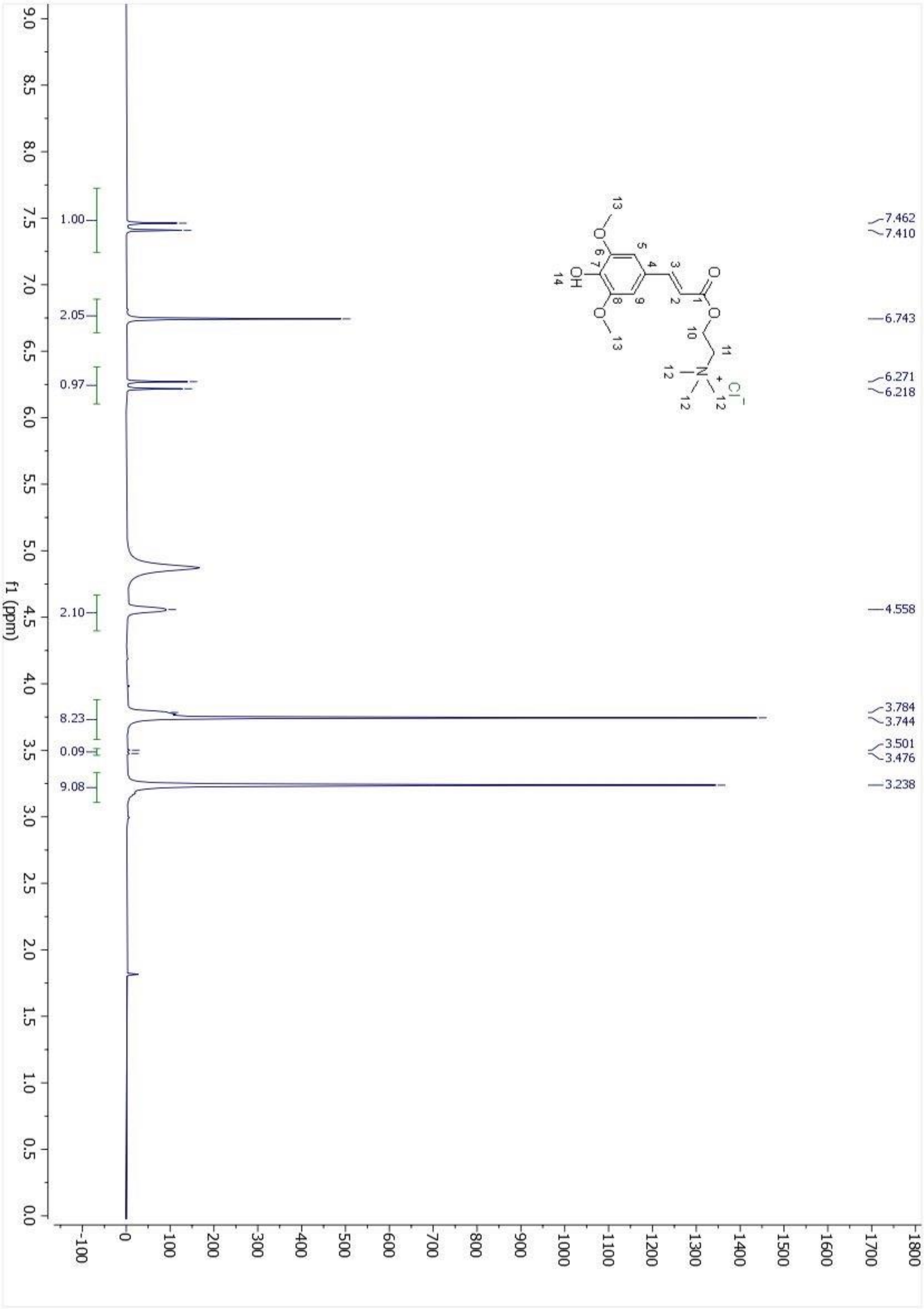

^{1}H NMR (300 MHz, CD_3OD): δ = 7.44 (d, J = 15.9 Hz, 1H, H-3), 6.74 (s, 2H, H-5 and 9), 6.24 (d, J = 15.9 Hz, ^{1}H, H-2), 4.56 (m, 2H, H-10), 3.78 (m, 2H, H-11), 3.74 (s, 6H, H-13), 3.24 (s, 9H, H-12).

Figure S2 : ^{13}C-NMR spectrum of sinapine

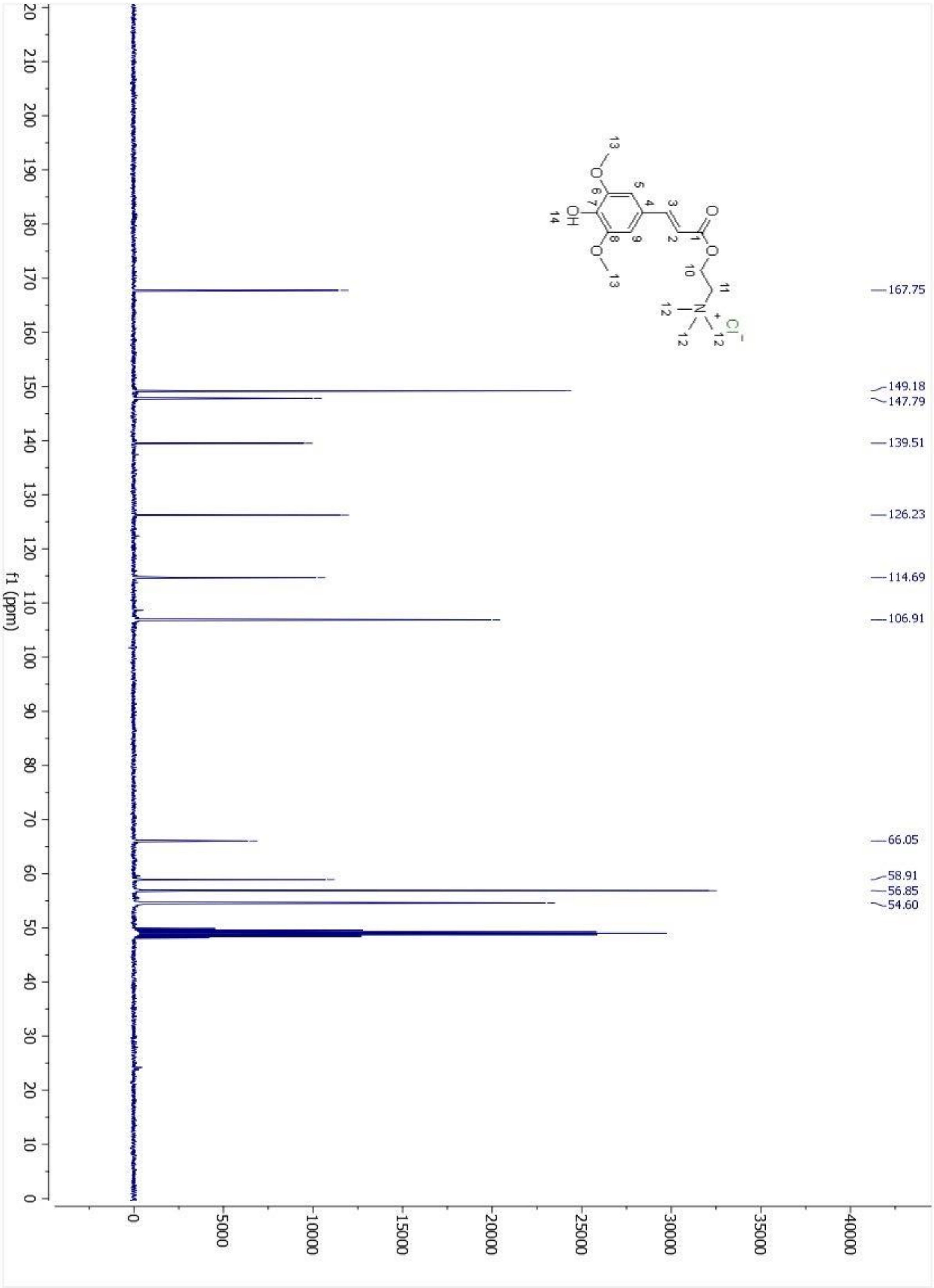

^{13}C NMR (75 MHz, CD_3OD): δ = 167.7 (s, C-1), 149.2 (s, C-6 and 8), 147.8 (d, C-3), 139.5 (s, C-7), 126.2 (s, C-4), 114.7 (d, C-2), 106.9 (d, C-5 and 9), 66.1 (t, C-11), 59.9 (t, C-10), 56.8 (q, C-12), 54.6 (q, C-13).

Figure S3 : ^{1}H-NMR spectrum of ethyl sinapate

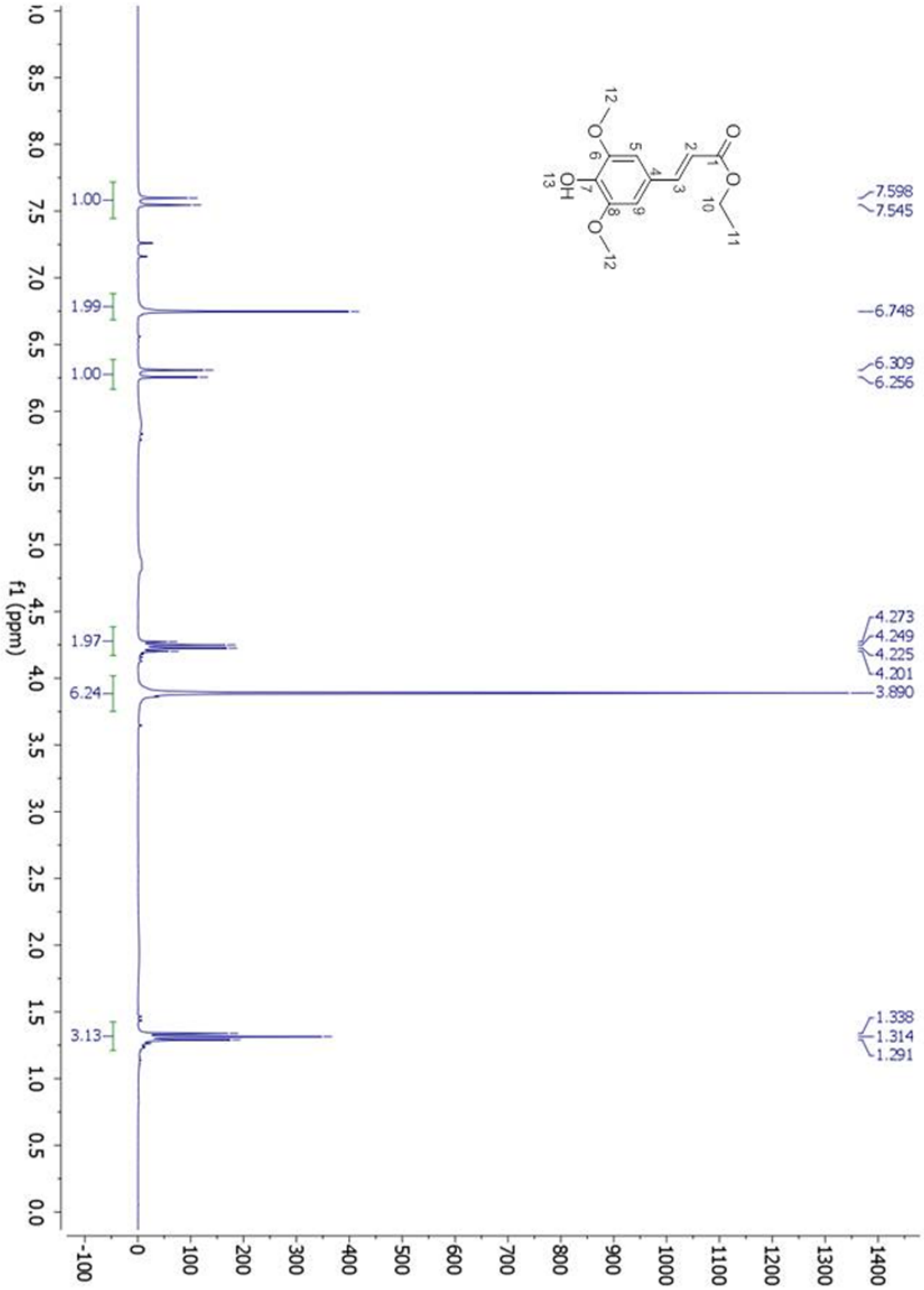

^{1}H NMR (300 MHz, $CDCl_3$): δ = 7.57 (d, J = 15.9 Hz, 1H, H-3), 6.75 (s, 2H, H-5 and 9), 6.28 (d, J = 15.9 Hz, 1H, H-2), 4.23 (q, J = 7.2 Hz, 2H, H-10), 3.89 (s, 6H, H-12), 1.31 (t, J = 7.2 Hz, 3H, H-11).

Figure S4 : ^{13}C-NMR spectrum of ethyl sinapate

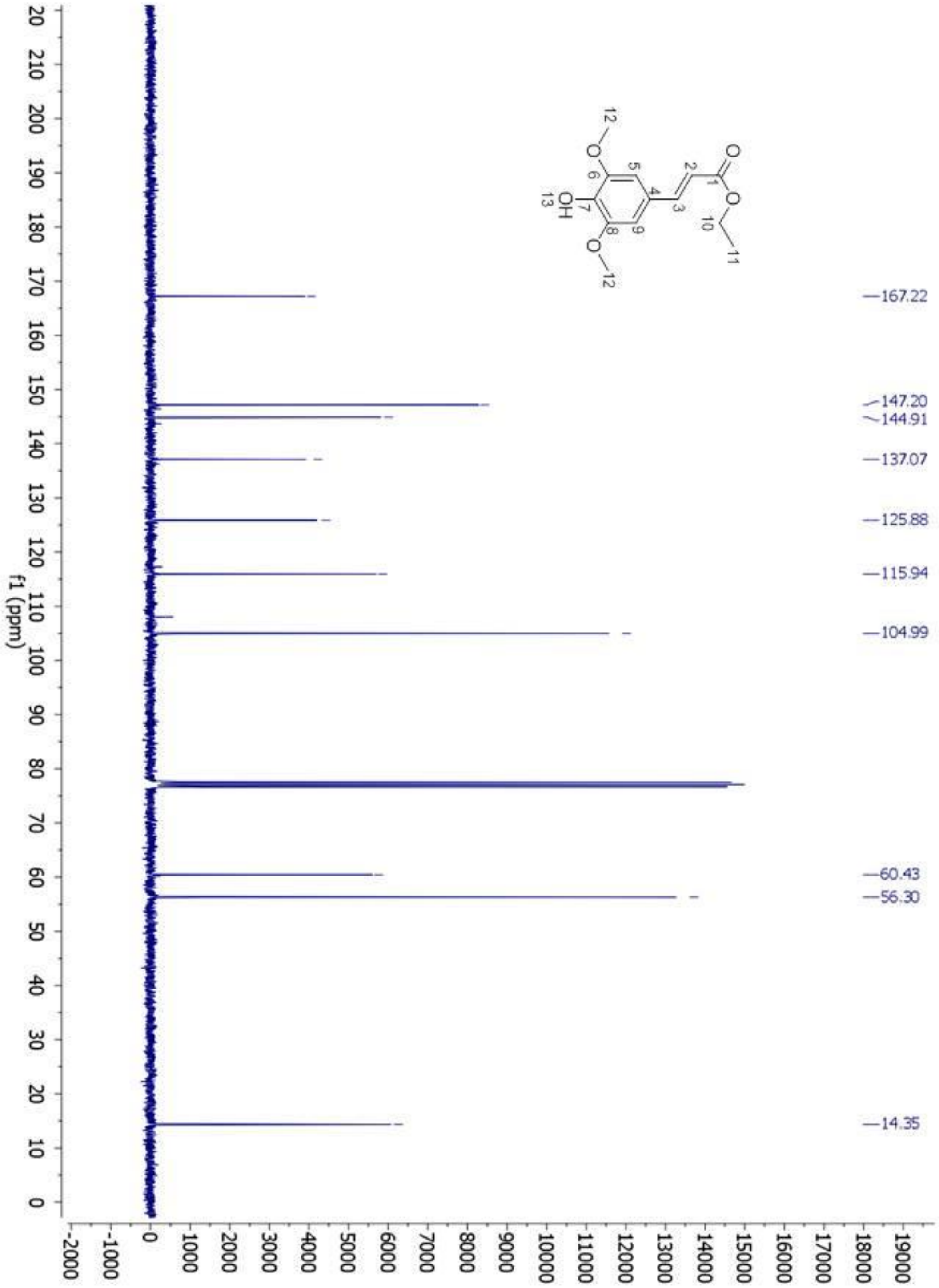

^{13}C NMR (75 MHz, $CDCl_3$): δ = 167.2 (s, C-1), 147.2 (s, C-6 and 8), 144.9 (d, C-3), 137.1 (s, C-7), 125.9 (s, C-4), 115.9 (d, C-2), 105.0 (d, C-5 and 9), 60.4 (t, C-10), 59.9, 56.3 (q, C-12), 14.4 (q, C-11).
